# Hydroxylated Metabolites of the Polybrominated Diphenyl Ether Mixture DE-71 Are Weak Estrogen Receptor-α Ligands

**DOI:** 10.1289/ehp.11343

**Published:** 2008-05-27

**Authors:** Minerva Mercado-Feliciano, Robert M. Bigsby

**Affiliations:** 1 Department of Pharmacology and Toxicology; 2 Department of Obstetrics and Gynecology, Indiana University School of Medicine, Indianapolis, Indiana, USA

**Keywords:** cytochrome P450, DE-71, endocrine disruptors, ERE-luciferase, estrogens, mice, ovariectomized, PBDEs, polybrominated diphenyl ethers

## Abstract

**Background:**

Polybrominated diphenyl ethers (PBDEs) are widely found in the environment and are suspected endocrine disruptors. We previously identified six hydroxylated metabolites of PBDE (OH-PBDEs) in treated mice.

**Objective:**

We tested the hypothesis that OH-PBDEs would interact with and alter activity of estrogen receptor-α (ER-α).

**Methods:**

We tested estrogenicity using two assays: ^3^H-estradiol (^3^H-E_2_) displacement from recombinant ER-α and induction of reporter gene (ERE-luciferase) in cultured cells. We incubated the PBDE mixture DE-71 with rat liver microsomes and tested the resultant metabolite mixture for estrogenic activity. We also determined relative estrogenic potential of individual hydroxylated PBDE congeners.

**Results:**

Reporter gene activity was increased by DE-71 that had been subjected to microsomal metabolism. DE-71 did not displace E_2_ from ER-α, but all six of the OH-PBDE metabolites did. *para*-Hydroxylated metabolites displayed a 10- to 30-fold higher affinity for ER-α compared with *ortho*-hydroxylated PBDEs, and one produced a maximal effect 30% higher than that produced by E_2_. Coadministration of E_2_ and DE-71, or certain of its metabolites, yielded reporter activity greater than either chemical alone. Two *ortho-*OH-PBDEs were antiestrogenic in the reporter assay.

**Conclusions:**

The observations—that the DE-71 mixture did not displace ^3^H-E_2_ from ER-α while the hydroxylated metabolites did—suggest that the weak estrogenic effects of DE-71 are due to metabolic activation of individual congeners. However, the behavior of DE-71 and its metabolites, when co-administered with E_2_, suggest a secondary, undetermined mechanism from classical ER-α activation.

DE-71 is a commercial mixture of mostly tetra- and penta-brominated diphenyl ethers (PBDEs), which has been used extensively as a flame retardant (Agency for Toxic Substances and Disease Registry 2004). DE-71 and other similar commercial mixtures (known collectively as pentaBDE) were used almost exclusively as flame retardants in flexible polyurethane foam, a major component of bed mattresses and upholstered products. Production of DE-71 ceased in 2004 (U.S. Environmental Protection Agency 2006), the same year that use of pentaBDE and other PBDE commercial mixtures was banned by the European Union (2003).

PBDEs are very stable compounds, and they are not chemically bonded to the material they are intended to protect from burning. As a result, they are widely found in environmental media (Hites 2004; Law et al. 2006) and can be found in human blood and milk (Furst 2006; Gomara et al. 2007; Lind et al. 2003; Main et al. 2007; Mazdai et al. 2003; Schecter et al. 2003). Some of the PBDE congeners most commonly found in human samples are BDE-47, BDE-99, and BDE-153 (Hites 2004; Gomara et al. 2007; Main et al. 2007; Mazdai et al. 2003). Two of these same congeners, the tetrabrominated BDE-47 and the pentabrominated BDE-99, are the main components of DE-71 (36% and 44% by weight, respectively); the hexabrominated BDE-153 is a minor component of DE-71 (4% by weight) (Qiu et al. 2007).

The prevalence of PBDEs in human tissue is of concern because these compounds are known to alter behavior, thyroid-hormone signaling, and sexual development in animals. Eriksson and colleagues found permanent aberrations in spontaneous behavior in rodents after developmental exposure to BDE-47 (Eriksson et al. 2001), BDE-99 (Eriksson et al. 2006; Viberg et al. 2004), or BDE-153 (Viberg et al. 2003). Serum thyroxine (T_4_) was significantly decreased in several different experimental models and by different pentaBDE mixtures: in rats exposed to DE-71 either prenatally or postnatally (Ellis-Hutchings et al. 2006; Stoker et al. 2004; Zhou et al. 2002); in American kestrels (*Falco sparverius*) exposed *in ovo* to a mixture of BDE-47, BDE-99, BDE-100 and BDE-153 (Fernie et al. 2005); and in adult female rats exposed to the commercial pentaBDE mixture Bromkal 70–5 DE (Darnerud et al. 2007). The effect of PBDEs on T_4_ levels may require metabolic activation because hydroxylated PBDEs, but not the non-hydroxylated congeners, are able to bind human transthyretin *in vitro* (Meerts et al. 2000). Other effects of pentaBDE mixtures or their congeners in experimental animals suggest estrogenic or antiandrogenic activity. In rats, developmental exposure to BDE-99 affected the regulation of estrogen target genes (Ceccatelli et al. 2006), impaired spermatogenesis (Kuriyama et al. 2005), and decreased circulating sex steroids and reduced anogenital distance in males (Lilienthal et al. 2006). Male rats exposed to DE-71 on postnatal days 23–53 had reduced seminal vesicle and ventral prostate weights and delayed puberty (Stoker et al. 2004).

PBDEs are suspected to behave as estrogens because of the similarity of their chemical structures and properties to other xeno-estrogens, mainly the polychlorinated biphenyls (PCBs) (Hooper and McDonald 2000; Meerts et al. 2001; Pijnenburg et al. 1995). We have shown that DE-71 has weak estrogenic activity *in vivo* and *in vitro* (Mercado-Feliciano and Bigsby 2008). Because hydroxylated metabolites of a structurally similar class of halogenated aromatic pollutants, the PCBs, exert estrogenic effects (Carpenter 2006; Vakharia and Gierthy 2000), it may be reasonable to expect that hydroxylated forms of PBDEs would also be estrogenic. Others have shown that some PBDE congeners and certain synthetically hydroxylated congeners could exert estrogenic effects in cultured cells (Hamers et al. 2006; Meerts et al. 2001). In a recent *in vivo* study, BDE-47 had uterotrophic effects in immature rats (Dang et al. 2007), suggesting *in vivo* activation of this otherwise nonestrogenic PBDE (Meerts et al. 2001).

We previously reported that DE-71 is metabolized in the mouse to produce hydroxylated metabolites (Qiu et al. 2007) and that it had mild estrogenic activity in the same animals (Mercado-Feliciano and Bigsby 2008). In the present study, our goal was to determine if DE-71 or its *in vivo* metabolites could induce estrogenic signaling though ER-α.

## Materials and Methods

### Test chemicals

We purchased dimethyl sulfoxide (DMSO) and estradiol [1, 3, 5(10)-estra-triene-3, 17β-diol; E_2_] from Sigma Chemical Co. (St. Louis, MO). The PBDE congener mixture DE-71 was a gift from the Great Lakes Chemical Corporation (West Lafayette, IN); the congener composition was previously described by Qiu et al. (2007). The individual hydroxylated metabolites of PBDE [4-OH-2, 2′, 4-tribromodiphenyl (4′-OH-BDE-17); 2′-OH - 2, 4, 4′ - tribromodiphenyl (2′-HO-BDE-28); 4-HO-2, 2′,3, 4′-tetra-bromodiphenyl (4-OH-BDE-42); 3-OH-2, 2′, 4, 4′-tetrabromodiphenyl (3-OH-BDE-47); 6-OH-2, 2′,4, 4′-tetra-bromodiphenyl (6-OH-BDE-47); and 4′-OH-2, 2′,4, 5′-tetrabromodiphenyl (4′-OH-BDE-49)] were synthesized as described by Marsh et al. (2004) and were gifts from G. Marsh (Stockholm University, Stockholm, Sweden). We purchased the brominated phenols 2, 4-dibromophenol (2, 4-DBP) and 2, 4, 5-tribromophenol (2, 4, 5-TBP) from Cambridge Isotope Laboratories (Cambridge, MA). DMSO was used as primary solvent for all chemicals, and the DMSO solutions were further diluted in cell culture media for treatments.

### Cells and culture conditions

MDA-MB-231 breast cancer cells (Cailleau et al. 1978) obtained from ATCC (American Type Culture Collection; Manassas, VA) and BG1Luc4E2 ovarian cancer cells, a gift from M. Denison (University of California, Davis, CA), were used in estrogen bioassays. BG1LucE2 cells are BG-1 ovarian cancer cells (Geisinger et al. 1989) stably transfected with an estrogen-responsive plasmid (Rogers and Denison 2000). Most cell culture media and supplements were purchased from Gibco/Invitrogen (Carlsbad, CA), except bovine growth serum (BGS; Hyclone, Logan, UT) and geneticin (G418; Sigma). Most charcoal-stripping reagents and endotoxin-free water were purchased from Sigma-Aldrich (St. Louis, MO) except Dulbecco’s phosphate-buffered saline (DPBS; Mediatech Inc., Herndon, VA). MDA-MB-231 cells were maintained in growth medium (GM): minimum essential media (MEM) supplemented with l-glutamine (2 mM), nonessential amino acids (0.1 mM), HEPES buffer (10 mM), 0.4 pg/mL insulin, and 5% vol/vol BGS]. BG1Luc4E2 cells were maintained in BG1-GM: alpha-MEM supplemented with HEPES buffer (10 mM), geneticin (0.4 g/L) and 10% vol/vol BGS. Basal medium (BM) for MDA-MB-231 cells consisted of a formulation similar to GM, except that phenol red–free MEM and 3% charcoal-stripped BGS were used. BM for BG1Luc4E2 cells (BG1-BM) consisted of phenol red–free Dulbecco’s modified Eagle media: Nutrient Mixture F12 (DMEM:F12; Gibco/Invitrogen) supplemented with HEPES buffer (10 mM) and 10% vol/vol charcoal-stripped BGS. BGS was stripped of estrogenic activity by methods described previously (Biswas and Vonderhaar 1987; Lippman et al. 1976).

### Estrogen response element-luciferase (ERE-luc) assays

We used two ERE-luciferase reporter gene systems: one transiently transfected and the other an established stably transfected cell line. For the transient system, ER-negative MDA-MB-231 breast cancer cells were plated in BM. Two days later cells were transfected using Tfx-20 (Promega, Madison, WI) with expression vectors for ER-α(HEG0; from P. Chambon, Université Louis Pasteur, Illkrich, France), the estrogen-responsive firefly luciferase reporter construct ERE2-pS2-luc (Long et al. 2001), and the control *Renilla* luciferase reporter construct pRL-TK (Promega). Cells were treated with test chemicals 1 hr after transfection, and assayed for luciferase activity after 18 hr. Results are expressed as the ratio of firefly luciferase to *Renilla* luciferase. For the stable reporter system, ER-positive BG1Luc4E2 ovarian cancer cells (Rogers and Denison 2000) were incubated in BG1-BM for 5 days before treatment; cells were then assayed for luciferase activity 18 hr after addition of the test compound.

### In vitro generation of microsomal metabolites

We incubated DE-71 or E_2_ with liver microsomes to obtain microsomal metabolites, following a procedure adapted from Bulger et al. (1978). Glcose-6-phosphate, glucose-6-phosphate dehydrogenase (G6PD) and β-nicotinamide adenine dinucleotide phosphate (oxidized form, NADP_+_) were purchased from Sigma-Aldrich. DE-71 (1 mM) or E_2_ (1 μM) were incubated for 24 hr with female rat liver microsomes (BD Biosciences Gentest, Woburn, MA) in a buffer that included an NADPH-generating system (50 mm Tris buffer, pH 7.5, 5 mM MgCl_2_, 12 mM glucose-6-phosphate, 0.4 mM NADP^+^, 2 units G6PD) in loose-capped tubes at 37°C with shaking. The incubation mixture was then centrifuged at 105,000 relative centrifugal force (RCF) at 4°C for 1 hr to remove microsomes. The hydroxylated organic fraction was extracted from the supernatant by solid-state extraction with ethanol elution using Sep-Pak Plus C18 cartridges (Waters Corp.; Milford, MA), then evaporated to dryness *in vacuo* and reconstituted in a volume of DMSO that would yield 10 mM PBDE or 10 μM estradiol, assuming 100% recovery. This extraction procedure was adapted from Yoshihara et al. (2004).

### Recombinant ER-α binding assay

Vehicle or test chemicals were incubated with 1 nM tritiated E_2_ (^3^H-E_2_; Amersham Biosciences, Buckinghamshire, UK) and 0.6 nM recombinant ER-α(Panvera/Invitrogen, Madison, WI) in TE buffer (10 mM Tris, 1 mM EDTA, pH 7.5) at 4°C overnight. Hydroxylapatite (60% in TE buffer) was added, mixed well, and incubated for 15 min at room temperature. The resulting slurry was washed three times by centrifugation at 3,000 RCF at 4°C with TE buffer changed. Bound ligand was extracted by incubation of the slurry with absolute ethanol at 30°C for 10 min. Tritium (^3^H) decay (counts per minute) was measured by liquid scintillation in a Beckman LS 5000 TD counter (Beckman-Coulter Inc., Fullerton, CA).

### Statistics

All statistics were performed using GraphPad Prism, version 3.0a, for Macintosh (GraphPad Software, San Diego CA). For each analysis, we determined whether groups had unequal variances by Bartlett’s test. Group averages with equal variances were compared to each other either by one-way analysis of variance (ANOVA) with Tukey post-test or by unpaired *t*-test as appropriate. Group averages with unequal variances were compared to each other by *t*-test with Welch’s correction. Groups treated with DE-71 or E_2_ alone were compared with vehicle controls, and groups cotreated with DE-71 and E_2_ were compared with controls treated with E_2_ alone. All values are expressed as mean ± SD or SE as indicated. We considered groups statistically different if *p* < 0.05 by ANOVA with Tukey posttest or *t*-test (two-tailed). Dose–response studies were also subjected to regression analysis using a sigmoidal curve fitting model:


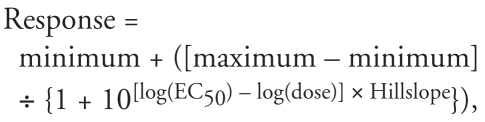


where EC_50_ is the median effective concentration. The modeled curve is shown in figures if *R*^2^ > 0.8.

## Results

### In vitro microsomal metabolism increases estrogenic character of DE-71

**Figure 1:**
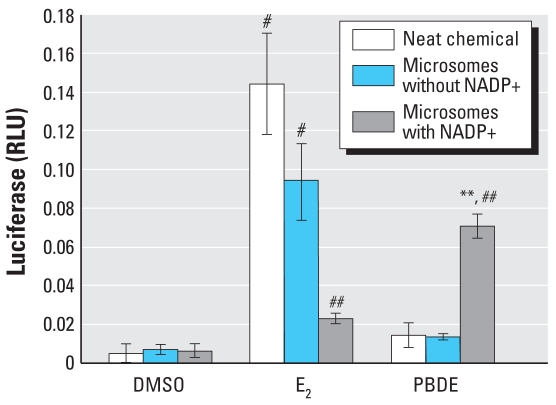
ERE-luciferase induction by microsomal metabolites of DE-71. Incubations with an incomplete NADPH generating system (lacking NADP+) were run in parallel and served as the negative control. The incubation products were tested in ERE-luciferase assays. One representative assay is shown; results are presented as mean ± SD (*n* = 4). The results are representative of three similar assays. **p < 0.01, and ^#^*p* < 0.001 compared with vehicle control. ^##^*p* < 0.001 compared with the same treatment without the complete NADPH generating system.

To determine whether microsomal metabolism could increase estrogenic activity, we incubated DE-71 with female rat microsomes, with or without a complete NADPH generating system. The incubation product was then tested in ERE-luciferase assays, using either the transient transfection system with MDA-MB-231 cells or the stably transfected ER-α–positive BG1Luc4E2 cells. At 10^−5^ M, DE-71 induced little ERE-luciferase activity when it was incubated in the buffer lacking an NADPH generating capacity (without NADP_+_; negative control), but the product of the complete incubation buffer produced a 4-fold increase in luciferase (Figure 1). Microsomal incubation decreased the activity of E_2_ by 78%, an expected result because hydroxylation decreases the activity of E_2_ (Martucci and Fishman 1993). Furthermore, the level of activity of the extract from the E_2_ incubated without NADP_+_ indicates that recovery of compound by the extraction methods used and in three separate microsomal incubation experiments was 66–98%.

### Phenolic metabolites found in blood of mice after treatment with DE-71

In a previous study (Qiu et al. 2007), we examined blood serum from BALB/c mice that had been treated with DE-71 for 34 days and quantified identifiable phenolic metabolites that included two brominated phenols and six hydroxylated brominated diphenyl ethers (Table 1). These findings raise the possibility that the estrogenic effects seen earlier in mice and in culture (Mercado-Feliciano and Bigsby 2008) were due to the metabolites and not necessarily the original DE-71 congeners.

### DE-71 and its phenolic metabolites activate the ERE

**Figure 2:**
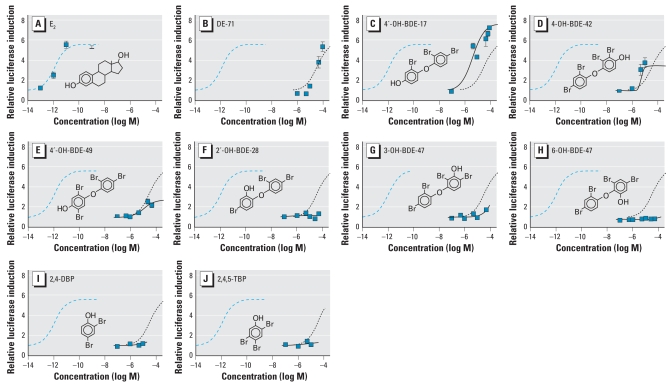
ERE-luciferase induction by E_2_ (*A*), DE-71 (*B*), and the OH-BDEs 4′-OH-BDE-17 (*C*), 4-OH-BDE-42 (*D*), 4′-OH-BDE-49 (*E*), 2′-OH-BDE-28 (*F*), 3-OH- BDE-47 (*G*), 6-OH-BDE-47 (*H*), 2, 4-DBP (*I*), and 2, 4, 5-TBP (*J*). See “Material and Methods” for details. Each curve for an OH-BDE represents the mean of 3–6 independent dose–response studies, except 6-OH-BDE-47, for which only 2 assays were performed. Curves for DE-71 and E_2_ are the mean of 12 and 8 independent dose–response studies, respectively. Error bars indicate SE. All values are normalized to vehicle control (DMSO = 1). Modeled data for E_2_ (dashed blue line) and DE-71 (dotted line) are shown in all OH-BDE charts for comparison. Modeled data for each OH-BDE are shown as a solid line.

E_2_ induces ERE-luciferase at concentrations above 10^−13^ M in the stably transfected BG1Luc3E2 reporter cell culture system (Figure 2), with an EC_50_ in the picomolar range (Table 2). Neat DE-71 and the metabolites found in mice were tested using BG1Luc3E2 cells to determine if they were able to activate ERE-mediated gene transcription (Figure 2), and their potencies and effectiveness were compared to that of E_2_ (Table 2). The potencies of each test compound were estimated by determining the compound’s own EC_50_ from its own maximal effect, and also by calculating its relative estrogenic potency determined from the concentration required to produce an effect equivalent to E_2_’s EC_50_.

DE-71 was able to significantly induce ERE-luciferase at test concentrations ≥ 5 × 10^−5^ M, reaching the same effectiveness as E_2_ at 10^−4^ M (Figure 2B). The calculated EC_50_ for DE-71 was 3.7 × 10^−5^ M. Because the maximal effect of DE-71 was very close to the maximal effect of E_2_, its EC_50_ (3.7 × 10^−5^ M) and its estrogen equivalency potency (EEP; 3.9 × 10^−5^ M) were similar. One metabolite, 4′-OH-BDE-17 was clearly more potent than DE-71, with an EC_50_ in the micromolar range (Table 2). 4′-OH-BDE-17 had a relative estrogenic potency approximately 10-fold that of DE-71, and it was more effective than DE-71 or E_2_, reaching an estimated maximal effect 30% higher than E_2_ (Figure 2C; Table 2). 4′-OH-BDE-49 had an EC_50_ similar to that of DE-71 but a much lower efficacy; its maximal effect did not even reach the EC_50_ for E_2_ (Figure 2E; Table 2). Another *para*-hydroxylated metabolite, 4-HO-BDE-42, appears to have been more potent than DE-71, but because of limited availability of this compound, the analysis was not carried out with a sufficient span of concentrations to allow an accurate estimate of the EC_50_ (Figure 2D).

### DE-71 phenolic metabolites displace ^3^H-E_2_ from ER-α

**Figure 3:**
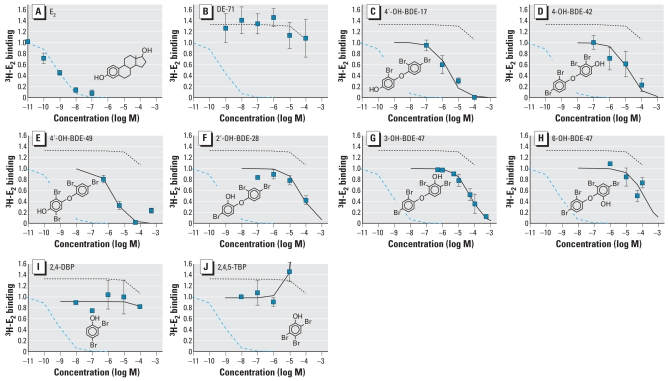
Displacement of 1 nM ^3^H-E_2_ from recombinant ER-α *in vitro* by E_2_ (*A*), DE-71 (*B*), and the OH-BDEs 4′-OH-BDE-17 (*C*), 4-OH-BDE-42 (*D*), 4′-OH-BDE-49 (*E*), 2′-OH-BDE-28 (*F*), 3-OH-BDE-47 (*G*), 6-OH-BDE-47 (*H*), 2, 4-DBP (*I*), and 2, 4, 5-TBP (*J*) found in mice (by the ER-α binding assay). Each curve for an OH-BDE represents the mean of 3–4 independent dose–response studies, except for 4-OH-BDE-49, 6-OH-BDE-47, and the bromophenols only 2 assays were performed. Curves for DE-71 and E_2_ are the mean of 6 and 11 independent dose–response studies, respectively. Error bars indicate SE. All values are normalized to vehicle control (DMSO = 1). Modeled data for E_2_ (dashed blue line) and DE-71 (dotted line) are shown in all OH-BDE charts for comparison. Modeled data for each OH-BDE are shown as a solid line.

We assessed the ability of the metabolites found in mouse blood to displace ^3^H-E_2_ from recombinant ER-α, and results are summarized in Figure 3 and Table 3. Neither the DE-71 mixture nor the bromophenol metabolites 2, 4-DBP and 2, 4, 5-TBP were able to displace ^3^H-E_2_ from ER-α (Figure 3B, I, J). All of the hydroxylated BDEs displaced ^3^H-E_2_ from ER-α, but their relative binding affinities were very low (Table 3). Of the OH-BDEs tested, the *para*-hydroxylated congeners (at either the 4 or 4′ position) had a higher affinity for the estrogen receptor than 2-, 3-, or 6-OH-BDEs. 4′-OH-BDE-17 and 4′-OH-BDE-49 were the most potent, with mean inhibitory concentrations (IC_50_) in the micromolar range (Figure 3C, D, E; Table 3). 6-OH-BDE-47, 3-OH-BDE-47, and 2′-OH-BDE-28 had IC_50_ values one order of magnitude higher than the *para*-OH-BDEs (Figure 3F, G, H; Table 3). In general, the potency of each OH BDE displacing ^3^H E_2_ from ER-α correlates with their ability to activate ERE-luciferase, and the congeners with the highest IC_50_ values induce very little (3 OH-BDE 47) or no significant ERE-luciferase activity (2′-OH-BDE-28 and 6-OH-BDE-47).

### DE-71 phenolic metabolites modify ERE activation by E_2_

**Figure 4:**
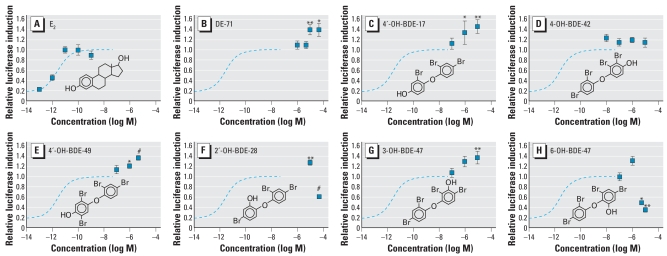
ERE-luciferase induction in BG1Luc4E2 cells after coteatment with E_2_. See “Material and Methods” for details. Squares indicate data for cotreatment with 10^−11^ M E_2_ and the specified chemical; data are normalized to the maximal effect of E_2_ (set at 1.0). Each curve is the mean of 3–5 independent dose–response studies, except for 2-OH-BDE28, 6-OH-BDE47, and 4-OH-BDE49 for which only 2 assays were performed. Error bars indicate SE. Modeled data for E_2_-only dose–response (blue dashed line) are shown in all charts for comparison. *p > 0.5; **p > 0.01; and ^#^*p* > 0.001 compared with the maximal effect of E_2_ alone.

Because DE-71 and several of its hydroxylated metabolites were able to either activate and/or displace ^3^H-E_2_ from ER-α, We cotreated BG1Luc3E2 cells with 10^−11^ M E_2_ and one of the chemicals of interest to determine if the PBDEs were able to modify E_2_-induced ERE-luciferase activity. The two bromophenol metabolites found in mice, 2, 4-DBP and 2, 4, 5-TBP, were not tested because they had no significant effect in either the ERE-induction or the ^3^H-E_2_ displacement assays. DE-71 induced ERE-luciferase beyond the maximal effect of E_2_ alone (Figure 4B). The same was true for 4′-OH-BDE-17 and 4′-OH-BDE-49 (Figure 4C, E). Another PBDE tested, 4-OH-BDE-42, appeared to induce ERE-luciferase above the E_2_ maximum, but the effect was not statistically significant (Figure 4D). The only *meta-*OH-PBDE tested, 3-OH-BDE-47, was not estrogenic by itself but was able to potentiate the ERE-luciferase induction of E_2_ (Figures 2G and 4G). The two *ortho*-hydroxylated BDE metabolites exhibited a biphasic dose–response curve in the E_2_ cotreatment assay. At high concentrations, 2′-OH-BDE-28 and 6-OH-BDE-47 were able to antagonize the effect of E_2_. 6-OH-BDE-47 was the more potent antagonist, showing an effect at 5 × 10^−6^M, whereas antagonism by 2′-OH-BDE-28 was observed only at 5 × 10^−5^ M (Figure 4F, H). Both of these metabolites also seem to potentiate the effect of 10^−11^ M E_2_ at lower concentrations in a manner similar to other PBDEs tested, although this effect was statistically significant only for 2′-OH-BDE-28. However, because only DE-71 and 2′-OH-BDE-28 were tested at a concentration of ≥5 × 10^−5^ M, it is possible that other hydroxylated PBDEs have the same biphasic behavior in the ERE-luciferase assay. The protein content of the culture wells at the time of harvest (not shown) indicated that there was no toxicity produced by 6-OH-BDE-47 or 2′-OH-BDE-28 at these concentrations (5–50 μM) when tested alone or in combination with E_2_. Interestingly, both OH-BDEs found to be estrogen antagonists were able to displace ^3^H-E_2_ from ER-α (Figure 3F, H), but when administered alone they did not induce significant ERE-luciferase (Figure 2F, H).

## Discussion

PBDEs are suspected to behave as estrogens because of the similarity of their chemical structure to other xenobiotics, mainly the PCBs (Crews et al. 1995; Ulbrich and Stahlmann 2004; Winneke et al. 2002). Furthermore, hydroxylated metabolites of PCBs have been shown to exert estrogenic effects (Blair et al. 2000; Kuiper et al. 1997). Therefore, it may be reasonable to expect that hydroxylated forms of PBDEs are also estrogenic. Our previous findings indicate that the PBDE mixture DE-71 is estrogenic *in vitro* and *in vivo*, although much less potent than E_2_ (Mercado-Feliciano and Bigsby 2008). Here, we show estrogenic and antiestrogenic effects of the phenolic metabolites of DE-71 by an interaction with ER-α.

Meerts et al. (2001) tested 17 PBDE congeners for estrogenic activity in an ERE-luciferase assay (ER-CALUX; Legler et al. 1999). Two of the congeners in DE-71, BDE-28 and BDE-100, were mildly estrogenic; BDE-100 was the most potent of the PBDEs although not the most effective. Using the same ER-CALUX bioassay, Hamers et al. (2006) showed weak estrogenic activity for the DE-71 congeners BDE-28, BDE-47, and BDE-100, but not for the pentaBDE mixture Bromkal 70-5DE. Results from Meerts et al. (2001) and Hamers et al. (2006) agree in the relative potency of these chemicals, and both groups agree that the EC_50_ for BDE-100 is in the micromolar range. In the present study we showed that DE-71 increased expression of ERE-luciferase reporter gene in BG1Luc3E2 cells, with potency similar to that of BDE-28 and BDE-100 in the ER-CALUX assay (Hamers et al. 2006; Meerts et al. 2001). The difference between our ERE-luciferase results and those of Hamers et al. (2006) can be accounted for by the fact that we tested higher concentrations. It may also be that the BG-1 cells we used and the T47D cells of the ER-CALUX assay differ in their ability to metabolically activate the various PBDE congeners.

In the present study, we observed metabolic activation of DE-71 to an estrogenic product *in vitro*, and previous experiments demonstrated that DE-71 congeners are hydroxylated in the mouse (Qui et al. 2007), a chemical modification that could increase their estrogenic activity. In the *in vitro* experiments, DE-71 was preincubated with rat liver microsomes, imitating the classical experiments by which Kupfer and Bulger demonstrated the metabolic activation of the proestrogen methoxychlor (Bulger et al. 1978; Kupfer and Bulger 1979). Preincubation of DE-71 with microsomes under enzyme-activating conditions increased its estrogenic activity. Mammalian liver microsomes are rich in cytochrome P450 (CYP450), a group of isoenzymes responsible for metabolism of many endogenous and exogenous chemicals including estrogens (reviewed by Bigsby et al. 2005). The biological activities of environmental chemicals have been found to be either increased or decreased by specific CYP450s (Goldstein and Faletto 1993), and some of these chemicals are known to be converted into estrogens by CYP450 metabolism (Bulger et al. 1978; Kohno et al. 2005; Kupfer and Bulger 1979; Morohoshi et al. 2005). Based on findings by Qui et al. (2007) and others (Malmberg et al. 2005; Marsh et al. 2006), the DE-71 congener BDE-47 seems to be the source of activated OH-PBDEs in laboratory rodents; BDE-47 itself has been found to have little estrogenic activity (Hamers et al. 2006; Meerts et al. 2001). Others have also found OH-PBDEs in wild marine animals that could be BDE-47 metabolites (Kelly 2006; Verreault et al. 2005; Verreault et al. 2007). However, the source of OH-BDEs in the marine environment can be both natural and anthropogenic, because some marine organisms produce natural brominated compounds (Vetter 2006).

We observed both estrogenic and anti-estrogenic effects in our study. We found that metabolites of DE-71 hydroxylated at the *ortho* position could act as antiestrogens. Although cotreatment with DE-71 produced a larger effect than the maximal response to E_2_ in the present study using a reporter gene assay, in another bioassay, based on MCF-7 cell proliferation, we observed both an agonist effect when DE-71 was administered alone and an antiestrogenic effect when coadministered with E_2_ (Mercado-Feliciano and Bigsby 2008). Because both MCF-7 cells and ovarian cancer cells express CYP450 enzymes (Deloia et al. 2008; Leung et al. 2007; Peters et al. 2004), it may be that the effects in cell proliferation and gene expression were due to metabolites generated in culture. The difference in responses between cell proliferation and reporter gene assays could result from generation of different metabolites in each assay due to differences in treatment duration (10 days vs. 18 hr, respectively) and/or the predominant CYP450 iso-enzyme activities in each kind of tissue (mammary vs. ovarian cancers). In addition, the estrogenic activity seen by other investigators for individual congeners in reporter gene bioassays (Hamers et al, 2006; Meerts et al. 2001) could be due to metabolic activation of those compounds. Thus, it is likely that the observed biological activity of DE-71 resulted from the sum of estrogenic and antiestrogenic activities of metabolites produced from individual BDE congeners.

Compounds may act as endocrine disruptors through a number of mechanisms, including indirectly by altering metabolism of endogenous hormones. Hamers et al. (2008) found that BDE-47 and several of the OH-PBDE metabolites that we found to have either estrogenic or antiestrogenic activity in culture also inhibit estrogen sulfotransferases (E2SULT) *in vitro*. Such an effect would translate into an increased activity of administered E_2_ and could explain the ability of 3-OH-BDE-47 to increase ERE-luciferase expression above the level induced by E_2_ alone when BG-1Luc4E2 cells are cotreated with both chemicals, even though 3-OH-BDE-47 does not induce ERE-luciferase by itself. However, the E_2_ dose used in cotreatment with 3-OH-BDE-47 was already high enough to reach the maximal effect in this system; therefore, it is unlikely that making more E_2_ available by inhibiting E2SULT would increase ERE-luciferase signaling. Furthermore, it is not known whether the BG-1Luc4E2 cells express E2SULT, and we observed no significant potentiation of the estrogenic effect by 4-OH-BDE-42, another metabolite that behaves as a potent inhibitor of E2SULT *in vitro*. A weaker E2SULT inhibitor, 6-OH-BDE-47, actually produced an antiestrogenic effect in our system. Thus, it is unlikely that altered E2SULT activity explains the additional estrogenic effect produced by 3-OH-BDE-47.

The levels of DE-71 needed to have estrogenic effects in our studies (micromolar range) are much higher than the highest concentrations found so far in human blood serum (0.1–5 nM; Mazdai et al. 2003). However, there is little current information on the levels of OH-PBDEs in human serum or the role that human enzymes (especially CYP450) may play in the formation of DE-71 metabolites. Rodent tissues do not have the same CYP450 activities as human tissues (Bogaards et al. 2000); therefore, the metabolites we found in mouse serum (Qui et al. 2007) or those found by Marsh et al. (2006) in rat feces may not be representative of metabolites formed in humans. Several of the PBDE metabolites we found in mice (4′-OH-BDE-17, 6-OH-BDE-47, 3-OH-BDE-47, and 4-OH-BDE-42) have been found in human serum samples from children working at a municipal waste disposal site (Athanasiadou et al. 2008) at much lower concentrations (< 0.1 nM) than are required to cause estrogenic effects in culture (≥ 1 μM) or associated with slight estrogenic effects in mice (> 40 nM; Table 1) following approximately 1 month of treatment (Mercado-Feliciano and Bigsby 2008). It is reasonable to expect that PBDE exposure for municipal waste workers is due in part to contact with DE-71 and other pentaBDE mixtures in discarded consumer products. However, Athanasioadou et al. (2008) found an additional PBDE metabolite in humans, 4-OH-BDE-90, that we did not observe in mice, and our own unpublished studies indicate that 5-OH-BDE-47 and 5′-OH-BDE-99 are major metabolites in human blood (Qui et al., in press); the estrogenic activity of these compounds has not been tested.

In summary, the observations that the DE-71 mixture does not displace ^3^H-E_2_ from ER-α—while the hydroxylated metabolites do—suggest that the weak estrogenic effects of DE-71 are due to metabolic activation of individual congeners. However, the behavior of DE-71 and some of its metabolites when coadministered with E_2_ suggest a secondary, undetermined mechanism of action different from classical ER-α activation.

## Figures and Tables

**Table 1:** Blood serum concentrations [μM (mean ± SE)] of phenolic metabolites found in mice after DE-71 treatment.

Compound	Vehicle control	DE-71
2,4-DBP	0.01 ± 0.02	0.29 ± 0.09
2,4, 5-TBP	0.001 ± 0.002	0.24 ± 0.09
4′-OH-BDE-17	ND	0.04 ± 0.02
2′-OH-BDE-28	0.0002 ± 0.0005	0.03 ± 0.01
4-OH-BDE-42	0.002 ± 0.01	0.36 ± 0.24
3-OH-BDE-47	ND	0.11 ± 0.05
6-OH-BDE-47	ND	0.04 ± 0.02
4′-OH-BDE-49	0.001 ± 0.002	0.08 ± 0.04
DE-71^a^	< 0.02	3.9 ± 0.8

ND, not detected. Mice were treated with 50 mg/kg/day DE-71 (per os) for 34 days. Concentrations were determined by gas chromatographic mass spectrometry analysis. Modified from Qiu et al. (2007).

a All non-OH congeners.

**Table 2:** Potency and efficacy estimates of DE-71 metabolites in the ERE-luciferase assay.

Compound	EC_50_^a^ (M)	EEP (M)^b^	Relative estrogen potency^c^ (ratio)	Fold-induction at EC_50_	Relative effect^d^ (ratio)
E_2_	1.2 × 10^−12^	—	1.00	3.3	1.00
DE-71	3.7 × 10^−5^	3.9 × 10^−5^	3.1 × 10^−8^	3.4	1.03
2, 4-DBP	No effect	—	—	—	—
2, 4, 5-TBP	No effect	—	—	—	—
4′-OH-BDE-17	4.7 × 10^−6^	3.5 × 10^−6^	3.4 × 10^−7^	4.3	1.30
2′-OH-BDE-28	NA	NR	—	—	—
4-OH-BDE-42	NA	NA	—	NA	NA
3-OH-BDE-47	NA	NR	—	NA	—
6-OH-BDE-47	No effect	—	—	—	—
4′-OH-BDE-49	1.3 × 10^−5^	NR	—	1.2	0.36

Abbreviations: NA, not available because effect was insufficient to calculate an EC_50_; NR, E_2_ EC_50_ not reached. All values were estimated from curves derived in Figure 2.

a Determined using the chemical’s own maximum effect set at 100%.

b Concentration inducing the same luciferase activity as the EC_50_ of E_2_.

c Ratio of the E_2_ EC_50_ to EEP.

d Test chemical-to-E_2_ ratio of luciferase induction at EC_50_.

**Table 3:** ER-α relative binding affinities.

Compound	IC_50_^a^	Relative affinity^b^(%)
E_2_	6.9 × 10^−10^	100
DE-71	No effect	—
2,4-DBP	No effect	—
2,4,5-TBP	No effect	—
4′-OH-BDE-17	2.1 × 10^−6^	0.03
2′-OH-BDE-28	6.0 × 10^−5^	0.001
4-OH-BDE-42	1.4 × 10^−5^	0.005
3-OH-BDE-47	5.2 × 10^−5^	0.001
6-OH-BDE-47	1.1 × 10^−5^	0.001
4′-OH-BDE-49	2.3 × 10^−6^	0.03

a The concentration of test compound yielding 50% displacement of ^3^H-E_2_ from receptor, calculated based on data shown in Figure 3.

b Relative affinity was calculated as E_2_ IC_50_ ÷ test chemical IC_50_ × 100.

## References

[b1-ehp-116-1315] Agency for Toxic Substances and Disease Registry (2004). Toxicological Profile for Polybrominated Biphenyls and Polybrominated Diphenyl Ethers (PBBs and PBDEs). http://www.atsdr.cdc.gov/toxprofiles/tp68.html.

[b2-ehp-116-1315] Athanasiadou M, Cuadra SN, Marsh G, Bergman A, Jakobsson K (2008). Polybrominated diphenyl ethers (PBDEs) and bioaccumulative hydroxylated PBDE metabolites in young humans from Managua, Nicaragua. Environ Health Perspect.

[b3-ehp-116-1315] Bigsby RM, Mercado-Feliciano M, Mubiru J, Naz RK (2005). Molecular mechanisms of endocrine disruption in estrogen dependent processes. Endocrine Disruptors: Effects on Male and Female Reproductive Systems.

[b4-ehp-116-1315] Biswas R, Vonderhaar BK (1987). Role of serum in the prolactin responsiveness of MCF-7 human breast cancer cells in long-term tissue culture. Cancer Res.

[b5-ehp-116-1315] Blair RM, Fang H, Branham WS, Hass BS, Dial SL, Moland CL (2000). The estrogen receptor relative binding affinities of 188 natural and xenochemicals: structural diversity of ligands. Toxicol Sci.

[b6-ehp-116-1315] Bogaards JJ, Bertrand M, Jackson P, Oudshoorn MJ, Weaver RJ, van Bladeren PJ (2000). Determining the best animal model for human cytochrome P450 activities: a comparison of mouse, rat, rabbit, dog, micropig, monkey and man. Xenobiotica.

[b7-ehp-116-1315] Bulger WH, Muccitelli RM, Kupfer D (1978). Studies on the in vivo and in vitro estrogenic activities of methoxychlor and its metabolites. Role of hepatic monooxygenase in methoxy-chlor activation. Biochem Pharmacol.

[b8-ehp-116-1315] Cailleau R, Olive M, Cruciger QV (1978). Long-term human breast carcinoma cell lines of metastatic origin: preliminary characterization. In Vitro.

[b9-ehp-116-1315] Carpenter DO (2006). Polychlorinated biphenyls (PCBs): routes of exposure and effects on human health. Rev Environ Health.

[b10-ehp-116-1315] Ceccatelli R, Faass O, Schlumpf M, Lichtensteiger W (2006). Gene expression and estrogen sensitivity in rat uterus after developmental exposure to the polybrominated diphenylether PBDE 99 and PCB. Toxicology.

[b11-ehp-116-1315] Crews D, Bergeron JM, McLachlan JA (1995). The role of estrogen in turtle sex determination and the effect of PCBs. Environ Health Perspect.

[b12-ehp-116-1315] Dang VH, Choi KC, Jeung EB (2007). Tetrabromodiphenyl ether (BDE 47) evokes estrogenicity and calbindin-D9k expression through an estrogen receptor-mediated pathway in the uterus of immature rats. Toxicol Sci.

[b13-ehp-116-1315] Darnerud PO, Aune M, Larsson L, Hallgren S (2007). Plasma PBDE and thyroxine levels in rats exposed to Bromkal or BDE-47. Chemosphere.

[b14-ehp-116-1315] Deloia JA, Zamboni WC, Jones JM, Strychor S, Kelley JL, Gallion HH (2008). Expression and activity of taxane-metobolizing enzymes in ovarian tumors. Gynecol Oncol.

[b15-ehp-116-1315] Ellis-Hutchings RG, Cherr GN, Hanna LA, Keen CL (2006). Polybrominated diphenyl ether (PBDE)-induced alterations in vitamin A and thyroid hormone concentrations in the rat during lactation and early postnatal development. Toxicol Appl Pharmacol.

[b16-ehp-116-1315] Eriksson P, Fischer C, Fredriksson A (2006). Polybrominated diphenyl ethers, a group of brominated flame retardants, can interact with polychlorinated biphenyls in enhancing developmental neurobehavioral defects. Toxicol Sci.

[b17-ehp-116-1315] Eriksson P, Jakobsson E, Fredriksson A (2001). Brominated flame retardants: a novel class of developmental neurotoxicants in our environment?. Environ Health Perspect.

[b18-ehp-116-1315] European Union (2003). Council Directive 2003/11/EC of the European Parliament.

[b19-ehp-116-1315] Fernie KJ, Shutt JL, Mayne G, Hoffman D, Letcher RJ, Drouillard KG (2005). Exposure to polybrominated diphenyl ethers (PBDEs): changes in thyroid, vitamin A, glutathione homeostasis, and oxidative stress in American kestrels (*Falco sparverius*). Toxicol Sci.

[b20-ehp-116-1315] Furst P (2006). Dioxins, polychlorinated biphenyls and other organohalogen compounds in human milk. Levels, correlations, trends and exposure through breastfeeding. Mol Nutr Food Res.

[b21-ehp-116-1315] Geisinger KR, Kute TE, Pettenati MJ, Welander CE, Dennard Y, Collins LA (1989). Characterization of a human ovarian carcinoma cell line with estrogen and progesterone receptors. Cancer.

[b22-ehp-116-1315] Goldstein JA, Faletto MB (1993). Advances in mechanisms of activation and deactivation of environmental chemicals. Environ Health Perspect.

[b23-ehp-116-1315] Gomara B, Herrero L, Ramos JJ, Mateo JR, Fernandez MA, Garcia JF (2007). Distribution of polybrominated diphenyl ethers in human umbilical cord serum, paternal serum, maternal serum, placentas, and breast milk from Madrid population, Spain. Environ Sci Technol.

[b24-ehp-116-1315] Hamers T, Kamstra JH, Sonneveld E, Murk AJ, Kester MH, Andersson PL (2006). In vitro profiling of the endocrine-disrupting potency of brominated flame retardants. Toxicol Sci.

[b25-ehp-116-1315] Hamers T, Kamstra JH, Sonneveld E, Murk AJ, Visser TJ, Van Velzen MJM (2008). Biotransformation of brominated flame retardants into potentially endocrine-disrupting metabolites with special attention to 2, 2′,4, 4′-tetrabromo-diphenyl ether (BDE-47). Mol Nutr Food Res.

[b26-ehp-116-1315] Hites RA (2004). Polybrominated diphenyl ethers in the environment and in people: a meta-analysis of concentrations. Environ Sci Technol.

[b27-ehp-116-1315] Hooper K, McDonald TA (2000). The PBDEs: an emerging environmental challenge and another reason for breast-milk monitoring programs. Environ Health Perspect.

[b28-ehp-116-1315] Kelly BC (2006). Bioaccumulation Potential of Organic Contaminants in an Artic Marine Food Web.

[b29-ehp-116-1315] Kohno Y, Kitamura S, Sanoh S, Sugihara K, Fujimoto N, Ohta S (2005). Metabolism of the alpha, beta-unsaturated ketones, chalcone and trans-4-phenyl-3-buten-2-one, by rat liver microsomes and estrogenic activity of the metabolites. Drug Metab Dispos.

[b30-ehp-116-1315] Kuiper GG, Carlsson B, Grandien K, Enmark E, Haggblad J, Nilsson S (1997). Comparison of the ligand binding specificity and transcript tissue distribution of estrogen receptors alpha and beta. Endocrinology.

[b31-ehp-116-1315] Kupfer D, Bulger W (1979). A novel in vitro method for demonstrating proestrogens. Metabolism of methoxychlor and *o, p*′DDT by liver microsomes in the presence of uteri and effects on intracellular distribution of estrogen receptors. Life Sci.

[b32-ehp-116-1315] Kuriyama SN, Talsness CE, Grote K, Chahoud I (2005). Developmental exposure to low dose PBDE 99: effects on male fertility and neurobehavior in rat offspring. Environ Health Perspect.

[b33-ehp-116-1315] Law RJ, Allchin CR, de Boer J, Covaci A, Herzke D, Lepom P (2006). Levels and trends of brominated flame retardants in the European environment. Chemosphere.

[b34-ehp-116-1315] Legler J, van den Brink CE, Brouwer A, Murk AJ, van der Saag PT, Vethaak AD (1999). Development of a stably transfected estrogen receptor-mediated luciferase reporter gene assay in the human T47D breast cancer cell line. Toxicol Sci.

[b35-ehp-116-1315] Leung HY, Wang Y, Leung LK (2007). Differential effect of over-expressing UGT1A1 and CYP1A1 on xenobiotic assault in MCF-7 cells. Toxicology.

[b36-ehp-116-1315] Lilienthal H, Hack A, Roth-Harer A, Grande SW, Talsness CE (2006). Effects of developmental exposure to 2, 2, 4, 4, 5-pentabromodiphenyl ether (PBDE-99) on sex steroids, sexual development, and sexually dimorphic behavior in rats. Environ Health Perspect.

[b37-ehp-116-1315] Lind Y, Darnerud PO, Atuma S, Aune M, Becker W, Bjerselius R (2003). Polybrominated diphenyl ethers in breast milk from Uppsala County, Sweden. Environ Res.

[b38-ehp-116-1315] Lippman M, Bolan G, Monaco M, Pinkus L, Engel L (1976). Model systems for the study of estrogen action in tissue culture. J Steroid Biochem.

[b39-ehp-116-1315] Long X, Gize EA, Nephew K, Bigsby RM (2001). Evidence for estrogenic contamination of the MAPK inhibitor PD98059. Endocrinology.

[b40-ehp-116-1315] Main KM, Kiviranta H, Virtanen HE, Sundqvist E, Tuomisto JT, Tuomisto J (2007). Flame retardants in placenta and breast milk and cryptorchidism in newborn boys. Environ Health Perspect.

[b41-ehp-116-1315] Malmberg T, Athanasiadou M, Marsh G, Brandt I, Bergman A (2005). Identification of hydroxylated polybrominated diphenyl ether metabolites in blood plasma from polybrominated diphenyl ether exposed rats. Environ Sci Technol.

[b42-ehp-116-1315] Marsh G, Athanasiadou M, Athanassiadis I, Sandholm A (2006). Identification of hydroxylated metabolites in 2,2′,4,4′-tetrabromodiphenyl ether exposed rats. Chemosphere.

[b43-ehp-116-1315] Marsh G, Athanasiadou M, Bergman A, Asplund L (2004). Identification of hydroxylated and methoxylated poly-brominated diphenyl ethers in Baltic Sea salmon (*Salmo salar*) blood. Environ Sci Technol.

[b44-ehp-116-1315] Martucci CP, Fishman J (1993). P450 enzymes of estrogen metabolism. Pharmacol Ther.

[b45-ehp-116-1315] Mazdai A, Dodder NG, Abernathy MP, Hites RA, Bigsby RM (2003). Polybrominated diphenyl ethers in maternal and fetal blood samples. Environ Health Perspect.

[b46-ehp-116-1315] Meerts IA, Letcher RJ, Hoving S, Marsh G, Bergman A, Lemmen JG (2001). *In vitro* estrogenicity of polybrominated diphenyl ethers, hydroxylated PDBEs, and polybrominated bisphenol A compounds. Environ Health Perspect.

[b47-ehp-116-1315] Meerts IA, van Zanden JJ, Luijks EA, van Leeuwen-Bol I, Marsh G, Jakobsson E (2000). Potent competitive interactions of some brominated flame retardants and related compounds with human transthyretin in vitro. Toxicol Sci.

[b48-ehp-116-1315] Mercado-Feliciano M, Bigsby RM (2008). The polybrominated diphenyl ether mixture DE-71 is mildly estrogenic. Environ Health Perspect.

[b49-ehp-116-1315] Morohoshi K, Yamamoto H, Kamata R, Shiraishi F, Koda T, Morita M (2005). Estrogenic activity of 37 components of commercial sunscreen lotions evaluated by in vitro assays. Toxicol In Vitro.

[b50-ehp-116-1315] Peters AK, van Londen K, Bergman A, Bohonowych J, Denison MS, van den Berg M (2004). Effects of polybrominated diphenyl ethers on basal and TCDD-induced ethoxyresorufin activity and cytochrome P450-1A1 expression in MCF-7, HepG2, and H4IIE cells. Toxicol Sci.

[b51-ehp-116-1315] Pijnenburg AM, Everts JW, de Boer J, Boon JP (1995). Polybrominated biphenyl and diphenylether flame retardants: analysis, toxicity, and environmental occurrence. Rev Environ Contam Toxicol.

[b52-ehp-116-1315] Qiu X, Bigsby RM, Hites RA Hydroxylated metabolites of polybrominated diphenyl ethers (PBDEs) in human blood samples from the United States. Environ Health Perspect.

[b53-ehp-116-1315] Qiu X, Mercado-Feliciano M, Bigsby RM, Hites RA (2007). Measurement of polybrominated diphenyl ethers and metabolites in mouse plasma after exposure to a commercial pentabromo diphenyl ether mixture. Environ Health Perspect.

[b54-ehp-116-1315] Rogers JM, Denison MS (2000). Recombinant cell bioassays for endocrine disruptors: development of a stably transfected human ovarian cell line for the detection of estrogenic and anti-estrogenic chemicals. In Vitro Mol Toxicol.

[b55-ehp-116-1315] Schecter A, Pavuk M, Papke O, Ryan JJ, Birnbaum L, Rosen R (2003). Polybrominated diphenyl ethers (PBDEs) in U.S. mothers’ milk. Environ Health Perspect.

[b56-ehp-116-1315] Stoker TE, Laws SC, Crofton KM, Hedge JM, Ferrell JM, Cooper RL (2004). Assessment of DE-71, a commercial polybrominated diphenyl ether (PBDE) mixture, in the EDSP male and female pubertal protocols. Toxicol Sci.

[b57-ehp-116-1315] Ulbrich B, Stahlmann R (2004). Developmental toxicity of polychlorinated biphenyls (PCBs): a systematic review of experimental data. Arch Toxicol.

[b58-ehp-116-1315] U.S. Environmental Protection Agency (2006). Polybrominated Diphenyl Ethers (PBDEs) Project Plan.

[b59-ehp-116-1315] Vakharia DD, Gierthy JF (2000). Use of a combined human liver microsome-estrogen receptor binding assay to assess potential estrogen modulating activity of PCB metabolites. Toxicol Lett.

[b60-ehp-116-1315] Verreault J, Gabrielsen GW, Chu S, Muir DC, Andersen M, Hamaed A (2005). Flame retardants and methoxylated and hydroxylated polybrominated diphenyl ethers in two Norwegian Arctic top predators: glaucous gulls and polar bears. Environ Sci Technol.

[b61-ehp-116-1315] Verreault J, Shahmiri S, Gabrielsen GW, Letcher RJ (2007). Organohalogen and metabolically-derived contaminants and associations with whole body constituents in Norwegian Arctic glaucous gulls. Environ Int.

[b62-ehp-116-1315] Vetter W (2006). Marine halogenated natural products of environmental relevance. Rev Environ Contam Toxicol.

[b63-ehp-116-1315] Viberg H, Fredriksson A, Eriksson P (2003). Neonatal exposure to polybrominated diphenyl ether (PBDE 153) disrupts spontaneous behaviour, impairs learning and memory, and decreases hippocampal cholinergic receptors in adult mice. Toxicol Appl Pharmacol.

[b64-ehp-116-1315] Viberg H, Fredriksson A, Eriksson P (2004). Investigations of strain and/or gender differences in developmental neurotoxic effects of polybrominated diphenyl ethers in mice. Toxicol Sci.

[b65-ehp-116-1315] Winneke G, Walkowiak J, Lilienthal H (2002). PCB-induced neurodevelopmental toxicity in human infants and its potential mediation by endocrine dysfunction. Toxicology.

[b66-ehp-116-1315] Yoshihara S, Mizutare T, Makishima M, Suzuki N, Fujimoto N, Igarashi K (2004). Potent estrogenic metabolites of bisphenol A and bisphenol B formed by rat liver S9 fraction: their structures and estrogenic potency. Toxicol Sci.

[b67-ehp-116-1315] Zhou T, Taylor MM, DeVito MJ, Crofton KM (2002). Developmental exposure to brominated diphenyl ethers results in thyroid hormone disruption. Toxicol Sci.

